# Continuous Spikes and Waves during Sleep: Electroclinical Presentation and Suggestions for Management

**DOI:** 10.1155/2013/583531

**Published:** 2013-08-06

**Authors:** Iván Sánchez Fernández, Kevin E. Chapman, Jurriaan M. Peters, Chellamani Harini, Alexander Rotenberg, Tobias Loddenkemper

**Affiliations:** ^1^Division of Epilepsy and Clinical Neurophysiology, Department of Neurology, Harvard Medical School, Boston Children's Hospital, Boston, MA 02115, USA; ^2^Department of Child Neurology, Hospital Sant Joan de Déu, Universidad de Barcelona, 08950 Barcelona, Spain; ^3^Department of Neurology, Children's Hospital Colorado, University of Colorado, Aurora, CO 80045, USA

## Abstract

Continuous spikes and waves during sleep (CSWS) is an epileptic encephalopathy characterized in most patients by (1) difficult to control seizures, (2) interictal epileptiform activity that becomes prominent during sleep leading to an electroencephalogram (EEG) pattern of electrical status epilepticus in sleep (ESES), and (3) neurocognitive regression. In this paper, we will summarize current epidemiological, clinical, and EEG knowledge on CSWS and will provide suggestions for treatment. CSWS typically presents with seizures around 2–4 years of age. Neurocognitive regression occurs around 5-6 years of age, and it is accompanied by subacute worsening of EEG abnormalities and seizures. At approximately 6–9 years of age, there is a gradual resolution of seizures and EEG abnormalities, but the neurocognitive deficits persist in most patients. The cause of CSWS is unknown, but early developmental lesions play a major role in approximately half of the patients, and genetic associations have recently been described. High-dose benzodiazepines and corticosteroids have been successfully used to treat clinical and electroencephalographic features. Corticosteroids are often reserved for refractory disease because of adverse events. Valproate, ethosuximide, levetiracetam, sulthiame, and lamotrigine have been also used with some success. Epilepsy surgery may be considered in a few selected patients.

## 1. Introduction

Continuous spikes and waves during sleep (CSWS) is an epileptic encephalopathy, that is, a condition in which the epileptic processes themselves are thought to contribute to the disturbance in cerebral function. CSWS is characterized by (1) seizures, (2) neurocognitive regression, and (3) an electroencephalography (EEG) pattern of electrical status epilepticus during sleep (ESES) [1–6]. ESES is characterized by marked sleep potentiation of epileptiform activity in the transition from wakefulness to sleep that leads to near-continuous bilateral (or occasionally lateralized) slow spikes and waves that occupy a significant proportion of nonrapid eye movement (non-REM) sleep [2, 4].

In this review, we summarize epidemiological, etiological, clinical, and EEG features in CSWS based on available data. We also suggest an approach to manage this syndrome and present it in the framework of a more general childhood seizure susceptibility syndrome.

## 2. Definitions

The terms “ESES,” “CSWS,” and “Landau-Kleffner syndrome” have been used interchangeably in the literature to refer to the EEG pattern of frequent spike-waves or to the associated epileptic encephalopathy with regression [6–8]. The EEG pattern and the associated epileptic encephalopathy are different concepts that might require differentiated names. A recent survey in North-America showed that the use of concepts in ESES and CSWS is very heterogeneous, and a common terminology is not available [9]. For the purposes of this review, we will use “ESES” when referring to the EEG pattern, “CSWS” when referring to the epileptic encephalopathy with global regression, and “Landau-Kleffner syndrome” (LKS) when referring to the epileptic encephalopathy with mainly language regression. We use this terminology in order to give unequivocal names to the different concepts, but we acknowledge that terminology is in progress, and it may change in the future.

The main focus of this review is on CSWS, a severe epileptic encephalopathy with (1) ESES on EEG, (2) seizures, and (3) developmental regression in, at least, two domains of development. Therefore, patients with developmental regression in mainly the language domain will be reviewed under the associated condition of “Landau-Kleffner syndrome” [4]. The borders between these entities are often difficult to delineate, and conditions may be considered as different presentations of the same electroclinical spectrum [10].

## 3. Epidemiology

CSWS is a rare condition that occurs only in children and adolescents. In an outpatient pediatric series, 1 out of 440 (0.2%) epileptic children had CSWS [11]. In tertiary pediatric epilepsy centers, around 0.5%–0.6% of patients were diagnosed with CSWS [12, 13]. Among children and adolescents undergoing epilepsy surgery for intractable seizures, about 1-2% of patients presented with CSWS [14, 15]. The exact frequency of CSWS is difficult to assess because of inconsistent inclusion criteria and study methodologies. Gender distribution in large series and reviews shows a male-to-female ratio of 4 : 3 to 3 : 2 [2, 16–21].

## 4. Clinical Features

CSWS is an age-related epileptic encephalopathy in which the clinical features evolve over time. The evolving nature of this syndrome allows the recognition of several clinical events: age at seizure onset, age at neurocognitive regression, and age at seizure freedom. These clinical events provide information to identify electroclinical stages in CSWS, namely, dormant stage (from birth to epilepsy onset), prodromal stage (from epilepsy onset to regression), acute stage (from regression to seizure freedom), and residual stage (after seizure freedom) [22–24].

### 4.1. Seizures

A typical child with CSWS initially has normal or moderately abnormal baseline development and then presents with seizures around 2–4 years of age. Patients with structural lesions of the brain tend to have seizures earlier (around 2 years of age) than patients without lesions (around 4 years of age) [4]. Seizures during the prodromal stage occur typically out of sleep and are frequently clonic or tonic-clonic unilateral seizures that rarely progress to unilateral status epilepticus [6, 16, 25]. During the prodromal stage, two or more seizure types are seen in only 20% of patients [16]. During the acute stage, there is a marked increase in the frequency and types of seizures, which become more difficult to control [2, 6, 16, 25, 26]. Unilateral seizures become rare, while atonic seizures appear and the motor components of the seizures lead to sudden falls. Atypical absence seizures increase in frequency and severity and may even evolve into absence status epilepticus [6, 16, 25]. The lack of tonic seizures has been classically considered a major feature of this syndrome and allows for differentiation from Lennox-Gastaut syndrome (LGS) [1, 6, 25].

### 4.2. Neurocognitive Regression

During the dormant stage, neurocognitive development is clinically normal in approximately two-thirds of cases [13, 25, 26]. A severe neurocognitive regression occurs around 5-6 years of age in most patients. Neurocognitive deterioration affects a wide spectrum of developmental and neurocognitive milestones in varying but often severe degrees. Regression domains include language, behavior, learning, memory, attention, social interactions, motor skills, and global intelligence [2, 3, 6, 8, 15, 17, 25, 27–30]. 

## 5. Electroencephalographic Features

During wakefulness, the EEG shows focal/multifocal spikes that increase in frequency during the acute stage. The hallmark EEG feature of CSWS is ESES. ESES is characterized by (1) marked potentiation of epileptiform discharges during non-REM sleep, leading to (2) a (near)-continuous, bilateral, or occasionally lateralized slow spikes and waves, (3) and these spikes and waves occur “during a significant proportion” of the non-REM sleep with a threshold ranging from 25% to 85% [1, 6, 15, 19–21, 25, 27, 31–35].

### 5.1. Evolution of ESES over Time

 Abnormal EEG findings are found during the prodromal period or even the dormant stage. Findings always include potentiation of spiking during non-REM sleep. During the acute stage, interictal epileptiform activity becomes much more frequent and severe with more widespread spikes of higher amplitude associated with a more abnormal background. During sleep, the EEG pattern presents as ESES [4]. ESES typically appears about 4–8 years of age and typically remits around 8-9 years of age [8, 17, 19, 35]. The age of detection of the ESES pattern on EEG varies widely in different studies and likely reflects the varied criteria of ESES used as well as the variations of time intervals in which EEGs are performed.

### 5.2. Cut-Off Value

 The initial definitions of ESES proposed that no less than 85% of the total duration of slow sleep should be occupied by slow spike-waves [6, 34]. This cut-off value has been followed by several authors [6–8, 15, 17, 21, 35–39], while other authors used lower cut-off percentages [19, 20, 40, 41]. The International League Against Epilepsy does not refer to any particular threshold and only requires that spike-waves be “continuous” and “diffuse” [1] leading to heterogeneous and variable use of cut-off values by the professionals caring for these children [9].

### 5.3. Quantification of Epileptiform Activity

**Figure 1:**
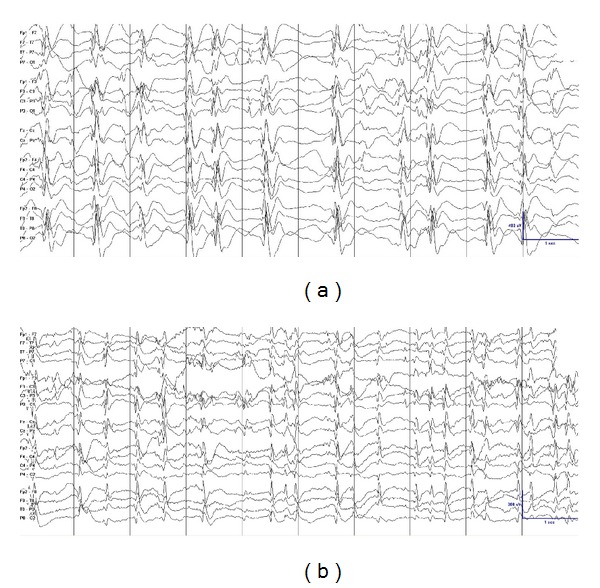
Different values in the quantification of the epileptiform activity result when using different methods of quantification. Epileptiform activity appears much more frequently in (b) than in (a). Both tracings have a 100% of epileptiform activity if the reader quantifies the epileptiform activity using spike percentage, that is, counting the percentage of 1-second bins occupied by spike-waves. However, if the reader quantifies the epileptiform activity using spike frequency, that is, the total number of individual spike-waves per unit of time (per 10 seconds in this page, per 100 seconds in a longer tracing), epileptiform activity is almost double in (b) than in (a). Note that the tracings have different voltage gains.

The classic measure for the quantification of epileptiform activity is the spike-wave index, expressed as the percentage of sleep occupied by spike-waves. While this percentage has been widely used, the exact method for calculating this value is often not specified [6, 7, 15, 17, 19, 21, 26, 35, 40]. A reproducible way to quantify epileptiform activity is to quantify the percentage of 1-second bins with at least one spike-wave in them, termed spike-wave percentage [24, 36]. Another reproducible method consists in counting the total number of spikes per unit of time, termed spike frequency [24]. A formal comparison between these two methods showed that spike frequency could better detect changes in epileptiform activity in those patients with very active epileptiform discharges (Figure 1) [24]. In addition, spike frequency lends itself better for automated quantification [42]. There is also no formal consensus on which portion of sleep is used for calculating the epileptiform activity with different periods of the night used by different authors [6, 19, 24, 35, 36, 43] and in clinical practice [9]. Regarding lateralized epileptiform activity, there is insufficient evidence to support that unilateral or focal discharges should be quantified differently than symmetric and bilateral discharges [20, 26, 39, 44].

## 6. Evolution over Time

CSWS evolves over time, and this evolution manifests in all three cardinal manifestations, including clinical seizures, EEG abnormalities, and neurocognitive regression. We therefore describe the evolution of this clinical presentation in these three categories.

### 6.1. Seizures

 Seizures almost always disappear with age, even in patients with a static or progressive encephalopathy [2, 3, 6, 25, 27, 29, 41, 45, 46]. The age of seizure freedom peaks around 6–9 years of age although data on this clinical event are scarce, and the range is wide [27, 29].

### 6.2. Electroencephalogram Features

 ESES progressively resolves with interictal epileptiform discharges during sleep substituted by a progressive return of the physiologic graphoelements and patterns of sleep. Typically, the resolution of the ESES pattern occurs around 8-9 years of age, in parallel with the timing of seizure freedom. However, ESES can persist and be very active for a period after seizure freedom [3, 24, 25, 29].

### 6.3. Neurocognitive Features

The initial regression ultimately leads to a plateau in development. Some patients present with moderate improvements after seizure freedom. However, most patients remain severely impaired [2, 3, 6, 47]. The impact of interictal spikes on neurocognitive features is a matter of debate, and it is not clear whether an increased amount of epileptiform activity is associated with a worse cognitive outcome [4, 48, 49].

## 7. Etiology and Pathophysiology

The exact cause of CSWS is unknown, but there are two factors that have been implicated. First, an association of CSWS with early developmental lesions of the brain has been shown. Second, an increasing number of genetic associations of unclear significance have also been described.

### 7.1. Early Developmental Lesions

**Figure 2:**
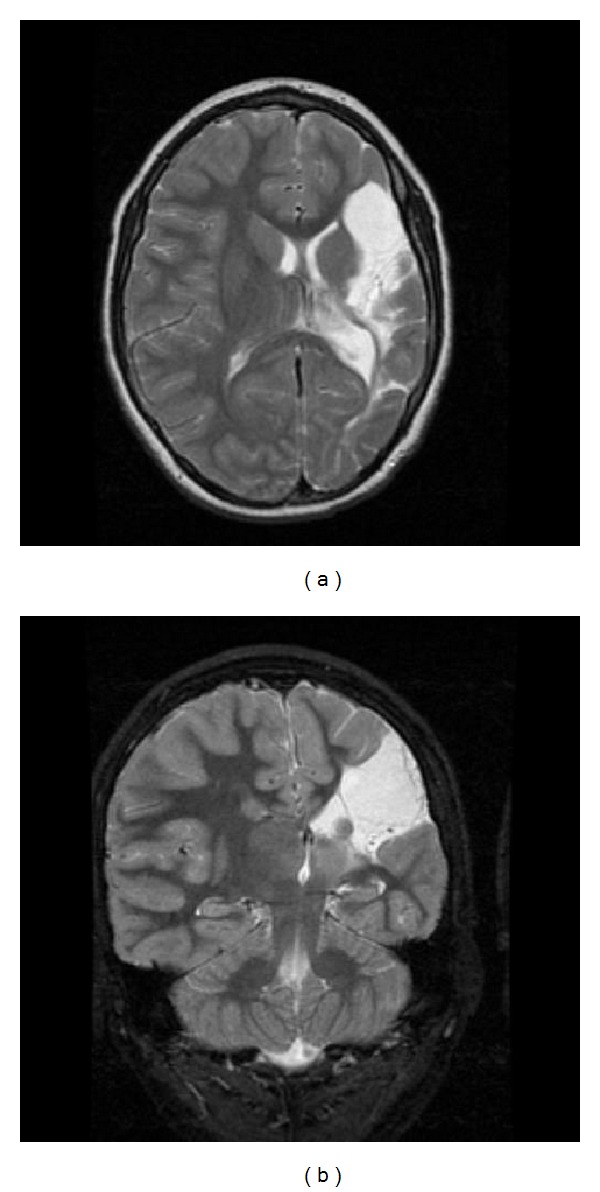
Early vascular lesions in patients with CSWS. Axial view T2 weighted in (a), coronal view T2 weighted in (b). Extensive cystic encephalomalacia affecting the left hemisphere in the distribution of the left middle cerebral artery consistent with a left middle cerebral artery infarct.

 Several case reports and small series described the association between patients with the ESES EEG pattern and early developmental lesions, such as malformations of cortical development [45], or vascular insults [50–52]. Larger series also support this association. In a study of 32 patients with prenatal or perinatal thalamic lesions, sleep potentiation of epileptiform activity occurred in 29 cases (90.6%) [27]. In two large series of patients with ESES, 33 out of 67 patients (49.3%) and 18 out of 44 (40.9%) patients had an early developmental lesion [20, 53]. While these lesions may not be specific for ESES, but for epilepsy in general, a recent series compared 100 patients with ESES and 47 patients with epilepsy without ESES. Patients with ESES had a higher frequency of early developmental lesions (48% versus 19.2%; *P* = 0.002) and a higher frequency of thalamic lesions (14% versus 2.1%; *P *= 0.037). These findings are consistent with other series suggesting that approximately 40–50% of patients with ESES had an early developmental insult [20, 53, 54], with a majority having perinatal lesions of vascular etiology [20, 53, 54]. Interestingly, some authors report that certain cortical malformations may also be related to early vascular insults [55]. In particular, early developmental lesions that involve the thalamus are strongly associated with CSWS (Figure 2) [54].

### 7.2. Genetic Factors

 Familial antecedents of seizures (including febrile seizures) are found in around 10–15% of patients with CSWS [6, 25]. Although genetic predisposition seems to play a minor role in CSWS, a growing number of case reports and small series describe associations with copy number variations and different mutations in several chromosomes (Table 1). The etiological role of these genetic factors in CSWS is largely undefined to date. It is likely that these genetic variants are associated not with CSWS *per se*, but with different neurological conditions that result in the final common pathway of CSWS. A similar theory is suggested for hypsarrhythmia in West syndrome.

### 7.3. Pathophysiology

 Animal models are providing insights into the basic pathophysiology of sleep-potentiated spiking [56]. Cortical lesions have been found to weaken the neurotransmission between corticothalamic neurons and the reticular nucleus of the thalamus without weakening the circuit between corticothalamic and thalamocortical neurons [57–59]. Therefore, reticular neurons do not have the normal loop interaction with the corticothalamic neurons that provides a feed-forward inhibition to thalamocortical neurons [57]. In contrast, a pathological loop with thalamocortical neurons is created which promotes a robust oscillatory network in the cortico-thalamo-cortical loop [57–59]. Breaking this pathological loop by selective inhibition of the thalamocortical neurons is a promising approach that has been found to work in an animal model [59]. The deficiency of the GluA4 AMPA receptor in a Gria4^−/−^ mouse model similarly weakens the normal output of the reticular neurons leading to the development of spike-wave discharges [57]. It can be hypothesized that lesions in the reticular nucleus of the thalamus may also lead to a potentiation of oscillatory discharges in the cortico-thalamo-cortical network. Supporting this hypothesis, marked sleep potentiation of epileptiform activity has been found in patients with early developmental lesions affecting the thalamus [27, 54]. The only study that evaluated the specific thalamic areas that were injured showed that the reticular nucleus was the most frequently affected structure and it was involved in 91% of the cases [27]. 

## 8. Management

### 8.1. To Treat or Not to Treat Epileptiform Activity

The relationship of epileptiform activity in the EEG with neuropsychological function is a matter of debate. Near-continuous epileptiform discharges are considered to be related to neurocognitive regression in CSWS [6, 48, 49, 60]. Many studies demonstrate that epileptiform activity is deleterious for learning and memory under certain experimental conditions [48, 49, 60–65], indirectly supporting the option of treatment. A recent study associated epileptiform activity during ESES with activation in the thalamocortical network and deactivation in the default mode network [38]. Since these networks seem prominent in neuropsychological processes and consolidation of memory traces during sleep, it is possible that epileptic spikes may contribute to regression in CSWS. On the other hand, the impact of interictal epileptiform activity on cognitive function may not be severe enough to serve as the sole explanation for the degree of neurocognitive regression [48, 65]. Many studies suggest that long-term neurocognitive function may significantly improve if epileptiform activity in the EEG can be reduced with antiepileptic drugs [3, 6, 7, 17, 19, 49], but this effect remains to be proven. Therefore, whether to treat epileptiform activity without a direct clinical correlate and, especially, to what extent to treat EEG findings is unclear. As a rule of thumb, we always treat the patient while considering the clinical presentation as a whole, not solely the EEG or other isolated laboratory values.

### 8.2. Modification of the Natural Course of the Disease by Treatment

Treatment goals of CSWS include not only improved seizure control, but also a reduction in EEG abnormalities and potentially improvement of neurocognitive function or at least prevention of further regression. There is evidence in the literature supporting a beneficial effect of treatment on seizure frequency and severity [7, 19, 26, 36, 66–70] and epileptiform activity [19, 21, 71, 72]. Several studies suggest that long-term neurocognitive function can significantly improve once epileptiform discharges are reduced, and this effect has been related to treatment with antiepileptic drugs [3, 6, 7, 17, 19, 73–77]. However, to date, there is no scientific evidence for or against treatment of interictal spikes.

### 8.3. Antiepileptic Drugs

The most common antiepileptic drugs used for CSWS include valproate, ethosuximide, and levetiracetam [78]. In a series of 15 patients with CSWS treated with high-dose valproate alone or with valproate and ethosuximide, 10 cases (67%) responded with long-term control of their epilepsy and partial recovery of cognitive function [19]. In a separate study, the combination of valproate and ethosuximide was effective in 2 additional patients [7]. In contrast, other series did not report similar improvements after treatment with comparable medication regimes. Valproate was reported as not effective in 28 patients [26]; valproate and benzodiazepines did not achieve any improvement in 7 patients and were associated with adverse behavioral reactions in 3 children [30], and several case reports describe no significant improvement with valproate [67, 68, 79]. Ethosuximide was also found to lack efficacy in 7 patients with CSWS [26] and to exert only a modest effect in 3 [67]. The efficacy of levetiracetam is supported by several case reports in the literature [26, 36, 66–68, 70]. The only placebo-controlled double-blind crossover study in patients with ESES showed that treatment with levetiracetam reduced epileptiform activity (from a spike index of 56 to 37) in a series of 18 patients, although 3 other patients discontinued treatment because of negative cognitive side effects [80]. Other drugs that have been reported as effective in small series include sulthiame [26, 81] and lamotrigine [45, 82]. Phenytoin, phenobarbital, and, especially, carbamazepine and oxcarbazepine are generally avoided because they have been associated with exacerbations of epileptiform discharges in patients with ESES [82–85].

### 8.4. High-Dose Benzodiazepines

Benzodiazepines have demonstrated efficacy in reducing epileptiform activity in the short term. Transitory resolution of the ESES pattern was observed after the administration of clonazepam [19, 21]. Diazepam has a shorter half-life than clonazepam, which can be advantageous in a condition such as CSWS where more severe epileptiform activity occurs during the night. In a series of 4 patients with CSWS refractory to valproate and ethosuximide, a short cycle of high-dose oral or intrarectal diazepam (0.5–1 mg/Kg per day for 6-7 days) was effective in the short term in two patients [19]. In a series of 15 patients with CSWS, all patients responded to the treatment with high-dose rectal diazepam [86]. High-dose oral diazepam (0.75–1 mg/Kg/day for 3 weeks) was also efficacious in 3 out of 8 (37.5%) patients, but the response was temporary [26]. In 29 patients with ESES and different clinical presentations, the mean epileptiform activity decreased from 77% to 41% after a nocturnal administration of 1 mg/Kg of oral diazepam [72]. This reduction in epileptiform activity persisted for some months [87], but whether this reduction in epileptiform activity is accompanied by a sustained improvement in clinical features remains to be proven. Other series show that 9 out of 10 patients did not respond to valproate and benzodiazepines, and 3 patients experienced an adverse behavioral reaction [30]. Adverse effects of high-dose diazepam treatment are generally considered mild and self-limited [72, 86], but severe behavioral disinhibition and even the need for discontinuation have also been described in few children [3, 87].

### 8.5. Immune Modulation Therapy

Corticosteroids and intravenous immunoglobulins have shown improvement in selected cases and, in some cases, lead to complete resolution of CSWS. Once CSWS is recognized, usually during the acute phase, corticosteroid treatment should be considered. In a series of 44 children with a pattern of ESES and clinical presentations of variable severity, prolonged corticosteroid treatment (hydrocortisone 5 mg/kg/day during the first month, 4 mg/kg/day during the second month, 3 mg/kg/day during the third month, and 2 mg/kg/day during the next 9 months, followed by slow withdrawal for a total treatment duration of 21 months) led to reductions of seizures or neuropsychological improvement in 34/44 (77.3%) cases, with 34 achieving complete control of seizures and normalization of EEG abnormalities in 21 patients. The long-term remission rate was 45% [53]. However, the inclusion of milder clinical presentations could make these results difficult to compare to other series where all or most patients had clear CSWS [53]. In another series, a positive response to different corticosteroids (prednisone, methylprednisolone, or adrenocorticotrophic hormone) was observed in 11 out of 17 patients with CSWS [26]. The intramuscular administration of 0.001–0.04 mg/kg/day of adrenocorticotrophic hormone was reported to be effective in 1 out of 4 patients [19]. The side effects of corticosteroid treatments usually limit its long-term use. Only a handful of patients treated with intravenous immunoglobulins have been reported in the literature. Intravenous immunoglobulin treatment was associated with improvements in 3 out of 9 patients with CSWS [26]. In another series, the neurocognitive function of 1 out of 3 patients with CSWS improved following the administration of intravenous immunoglobulins [88]. However, there is probably a publication bias of positive results, and the high cost and risk of complications associated with immunoglobulins make their role in the treatment of CSWS unclear.

### 8.6. Surgical Treatment

 Although classically epilepsy surgery was performed on patients with focal discharges, it has also been successfully applied to select patients with generalized discharges [89, 90]. Some patients with CSWS may also benefit from surgical treatment. Surgical interventions include multiple subpial transections (MSTs), focal resective surgery of the epileptogenic zone, hemispherectomy and corpus callosotomy. MST consists of multiple small superficial parallel cuts in the cortex that theoretically severs only the local corticocortical connections in an attempt to disrupt local epileptic circuitry without altering the vertical neural columns and their function. It has been reported to lead to recovery of age-appropriate speech in 7 patients out of a series of 14 patients with Landau-Kleffner syndrome [89], whereas a less dramatic language improvement was found in other series [91, 92]. Two patients with CSWS secondary to neonatal stroke markedly improved after hemispherectomy [93]. In another study, two patients with CSWS secondary to early developmental lesions in the thalamus became seizure-free after a hemispherectomy in one and after an extensive corticectomy around a large porencephalic cyst in the other [27]. A study evaluated epilepsy surgery in 13 patients with CSWS secondary to different early developmental lesions who underwent various surgical procedures including anterior callosotomy (6 patients), complete callosotomy (3 patients), hemispherectomy (2 patients), and lobar resection (2 patients). Subjects achieved an overall improvement in seizure control and EEG features in most patients [8]. 

Improvements may be related to the type of surgery performed. The cognitive deterioration may be halted in most patients; however, while there was some cognitive recovery, patients did not return to baseline. In a series of 8 patients with CSWS secondary to perinatal infarction (7 patients) and a malformation of cortical development (1 patient), 6 patients underwent a hemispherectomy, and 2 underwent focal resection. Results included disappearance of the pattern of ESES (all 8 patients), seizure freedom (6 patients), marked improvement in seizure control (2 patients), and an overall improvement in cognitive function (in 3 out of 5 patients with neuropsychological evaluation) [15]. Patients with CSWS should undergo epilepsy surgery only after a careful evaluation of potential benefits and risks in the individual patient. A tendency toward neurocognitive improvement was found in 3 out of 5 patients with CSWS after epilepsy surgery [15]. However, data on the long-term neurocognitive outcome of surgically managed CSWS patients are not available.

### 8.7. General Suggestions for Managing Patients with CSWS (Figure 3)

**Figure 3:**
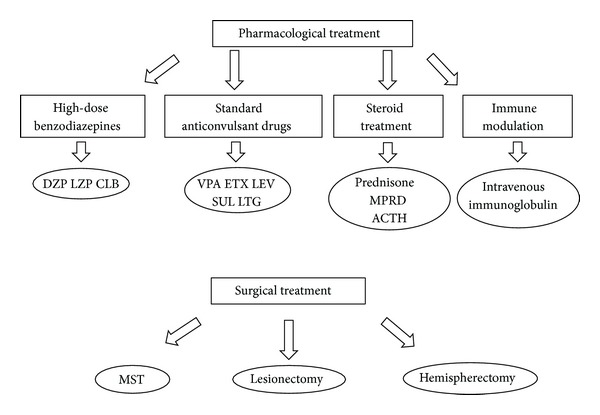
Options for the management of patients with CSWS. Options for chronic management are high-dose benzodiazepines, standard antiepileptic drugs in different combinations, and corticosteroids and immune-modulating agents. These options are considered as first choices by different authors, although standard antiepileptic drugs are generally used before the recognition of CSWS. Epilepsy surgery is reserved for few selected refractory cases. Legend: ACTH: adrenocorticotrophic hormone. CLB: clobazam. DZP: diazepam. ETX: ethosuximide. LEV: levetiracetam. LTG: lamotrigine. LZP: lorazepam. MPRD: methylprednisolone. MST: multiple subpial transection. SUL: sulthiame. VPA: valproate.

Current literature does not permit the development of an evidence-based management approach to CSWS. Most of the drugs used for CSWS are selected based on individual experience, case reports, or small case series that claim efficacy for a specific drug. Responses to treatment in uncontrolled case reports or case series should be interpreted with caution as any treatment for a disorder with a fluctuating natural course tends to be initiated at the peak of severity, so that some improvement can be attributed to the natural fluctuations of the disease. In addition, other series report a lack of efficacy for commonly used treatment options for CSWS. There is no evidence on the efficacy of the ketogenic diet in patients with CSWS. Here, we provide a practical treatment approach based on case series in the literature (Figure 3). In practice, most patients with CSWS were already on some standard antiepileptic drug (valproate, levetiracetam, or similar) when their seizures first began and before their condition was recognized as CSWS. Once in the acute phase, standard antiepileptic drugs, corticosteroids, and benzodiazepines can be considered as first choices depending on the particular patient and the familiarity of the physician with these drugs. Several groups have reported the usefulness of benzodiazepines [19, 26, 72, 86], and a frequent protocol used at our institutions is nocturnal Diazepam 1 mg/Kg during the first night followed by 0.5 mg/Kg every following night for 1–3 months [87]. For the chronic management of CSWS and particularly for seizure control standard antiepileptic drugs such as valproate, ethosuximide, levetiracetam, sulthiame, and lamotrigine are frequently used. Polytherapy is often needed. Medication selection should be guided by presenting seizure types [7, 19, 26, 30, 36, 45, 66–68, 70, 78–82]. Other options include treatment with corticosteroids, adrenocorticotrophic hormone, or intravenous immunoglobulin [19, 26, 53, 88]. Epilepsy surgery should be considered, especially in patients with an early unilateral developmental lesion, even when the epileptiform activity on EEG is generalized [8, 15, 27, 89–93]. For the acute control of very active nighttime epileptiform discharges, high-dose benzodiazepines have been used over a period of a few months [19, 21, 26, 72, 86]. While adequate control of seizures improves the quality of life of the patients and should be pursued, it is unknown how aggressively interictal epileptiform activity in relationship with neurocognitive regression should be treated. Only prospective studies that correlate the response to treatment of interictal epileptiform activity with the improvement in neurocognitive function will be able to answer that relevant question.

## 9. Related Conditions

ESES, the EEG pattern that characterizes CSWS, can be found in other electroclinical conditions. CSWS might represent the most severe end of a continuum in which Landau-Kleffner syndrome would be an intermediate condition and “benign” focal epilepsy syndromes of childhood would be at the most benign end of the spectrum.

### 9.1. Landau-Kleffner Syndrome

It is an age-related epileptic encephalopathy where regression occurs mainly in the language spectrum and the EEG abnormalities are more centered around the temporal-parietal regions [94]. Seizures are not a prominent part of this syndrome, and they are either infrequent or do not even occur in 20–30% of cases [3, 74]. Contrary to CSWS, structural brain lesions in LKS are an exception to the rule [94]. Most antiepileptic drugs are effective for seizure control in LKS [7, 18, 21]. Corticosteroids have been reported to markedly improve the evolution of the disease [18, 53, 77], and intravenous immunoglobulins demonstrated promising results in very few cases, although immunoglobulins are expensive and associated with potentially serious side effects [88, 95–99]. Resective surgery is not an option because the focus of epileptiform activity frequently involves eloquent cortex, including language areas. The technique of multiple subpial transections has led to variable results [91, 92, 100]. Similar to CSWS, seizures and EEG abnormalities normalize over time, but most patients do not recover their baseline language status [76].

### 9.2. “Benign” Pediatric Focal Epileptic Syndromes

They include “Benign” epilepsy of childhood with central-temporal spikes, Panayiotopoulos syndrome, and Gastaut-type late-onset childhood occipital epilepsy. These syndromes share features such as a strong genetic predisposition, age-related appearance and disappearance of electroclinical manifestations, and a relatively “benign” clinical course. As in the previous syndromes, interictal epileptiform activity may be disproportionately severe in comparison with the seizure correlation. Neurocognitive dysfunction, if present, is mild. The individual description of each particular syndrome is beyond the scope of this review and can be found elsewhere [101, 102]. Because of their main features, “benign” pediatric focal epileptic syndromes may be considered as part of the electroclinical spectrum of CSWS [101, 102].

### 9.3. Seizure (or Spikes) Susceptibility Syndrome

 CSWS, Landau-Kleffner syndrome and “benign” pediatric focal epilepsy syndromes share a series of common features: (1) an electroclinical syndrome consisting of seizures, interictal epileptiform activity, and neuropsychological deficits of different severities, (2) an age-related evolution with onset in early childhood and spontaneous improvement before puberty, (3) interictal epileptiform activity becomes markedly potentiated during non-REM sleep, (4) interictal epileptiform activity is disproportionately severe in comparison with the seizure correlate, and (5) interictal epileptiform activity frequently persists after seizure freedom. Overlap between these clinical presentations has led to the hypothesis of a common seizure susceptibility syndrome. In this syndrome, the different electroclinical presentations reflect different severities of a common underlying pathophysiology, similar to what happens with the different clinical presentations of hypsarrhythmia. A genetic or acquired disruption of the neural networks early in development would create hyperexcitable neural networks [57, 58] that, depending on its severity and localization, could manifest as different electroclinical presentations in the spectrum [27, 54, 56, 101, 103].

## 10. Conclusions

CSWS is an age-related epileptic encephalopathy that represents the most severe end of the childhood seizure susceptibility syndrome. Its characterizing features are (1) seizures, (2) interictal epileptiform activity that becomes prominent during sleep leading to the electroencephalogram pattern of ESES, and (3) neurocognitive regression. The etiology of CSWS is unknown, but early developmental lesions play a major role in around half of the cases. The neurocognitive outcome is generally poor, and it is currently unknown whether treatment can modify it. High-dose benzodiazepines have been used successfully to decrease very active epileptiform discharges. Polytherapy with combinations of valproate, ethosuximide, levetiracetam, sulthiame or lamotrigine, and corticosteroids is frequently used. Epilepsy surgery can be considered in a few very selected number of patients. A better understanding of the response to treatment, the electroclinical spectrum, and the underlying pathophysiology may allow for the development of an evidence-based management approach in the future.

## Figures and Tables

**Table 1:** Genetic factors that have been described in association with CSWS.

Study	Type of study	Association
Beaumanoir et al., 1995 [104]	Case report	CSWS in two monozygotic twins

Praline et al., 2006 [105]	Case report	Two siblings with ESES and different clinical presentations

Verhoeven et al., 2012 [106]	Case report	One patient with CSWS and dysmorphic features carried a de novo 8q12.3q13.2 microdeletion

Godfraind et al., 2008 [107]	Case report	One patient with CSWS carried a G392R mutation in neuroserpin of probable pathogenic significance (the mutation led to a progressive neurodegenerative disease and CSWS)

Nakayama et al., 2012 [108]	Case series (2 patients with CSWS)	Two patients with CSWS and dysmorphic features carried an unbalanced translocation between 8p and 9p

Broli et al., 2011 and Giorda et al., 2009 [109, 110]	Case series (2400 subjects with isolated or syndromic intellectual disability)	Five patients with CSWS carried a Xp11.22-p11.23 duplication

Kevelam et al., 2012 [40]	Case series (13 children with ESES and different clinical presentations)	Two patients with CSWS carried copy number variations in CHRNA7 and PCYT1B genes of probable pathogenic significance

Mefford et al., 2011 [111]	Case series (315 patients with different epileptic encephalopathies, 29 had CSWS or Landau-Kleffner syndrome)	One patient with CSWS carried a copy number variant in the DOK5 gene of uncertain pathogenic significance

Reutlinger et al., 2010 [112]	Case series (3 patients with ESES and different clinical presentations)	Three patients with ESES and different clinical presentations and dysmorphic features carried a deletion in 16p13.2p13.13

Atkins and Nikanorova, 2011 [66]	Case series (20 patients with ESES and different clinical presentations)	One patient with ESES (no further details on clinical presentation) carried a partial trisomy 13/21

Legend: CSWS: continuous spikes and waves during sleep. ESES: electrical status epilepticus in sleep.
